# Good Living and the Brain

**Published:** 1888-12

**Authors:** 


### GOOD LIVING AND THE BRAIN.

It is a common opinion that good, or rather high living, is the principal cause of dyspepsia ; but while the quantity and quality of our food and the manner of eating it doubtless has much to do with the behavior of our stomachs, the state of our brains has fully as much or more influence. Some of the most healthy people eat as much of any and all things as they desire without consulting any dietetic rules ; and others who pay great attention to their diet are the victims of dyspepsia ; but in most of these cases it will be found that these people take but little exercise and overwork their brains in reading, writing and the anxious pursuits of business. They sit down to a meal with minds absorbed and preoccupied to such an extent that they cannot tell five minutes after eating what they ate, or whether they had eaten at all ; and then they rush off to their business or literary work, thus diverting from the stomach to the brain the energy which should be concentrated on the stomach for the performance of its digestive functions. The brain being the source and fountain of all nervous influence, the organ which controls all the functions of the body, it is not strange that people should be dyspeptic when the blood and nerve forces which should be concentrated to the stomach are diverted to the brain.

One of the most important rules for the avoidance and cure of dyspepsia is, to eat with a quiet mind, and then to rest quietly for an hour or two after eating.

This simple rule, with a reasonable regard to the quantity and quality of food, will cure many cases of dyspepsia. It has been truly said that head workers need more rest than hand workers ; and that three hours of hard brain work are more exhaustive to the nervous energies than a whole day of ordinary manual labor. Therefore, above everything else, brain workers need sleep, sleep through the whole night and a nap in the day, especially after dinner. This is in accordance with nature as is manifested by the habits of the lower animals, which lie down and sleep after eating.

